# Annotations

**Published:** 1904-07-16

**Authors:** 


### ANNOTATIONS.

### Expert Witnesses.

The relationship of a person competent to give skilled evidence in a court of law to a subpœna summoning him to attend a trial as an expert witness has always been a somewhat uncertain one. Of course, if, as commonly occurs to the medical practitioner, the individual subpœnaed has a personal knowledge of the facts of the case in question he has no option. In such circumstances he is, at least in part, a "common" witness and must necessarily both obey the summons and answer questions dealing with matters of opinion as well as with matters of fact. The uncertainty, so far as it exists, is attached to the pure expert, that is to one whose technical knowledge entitles him to express a skilled opinion on the significance and value of facts related by others. Can anyone in this position be compelled, whether he will or no, to attend a court of law during a particular trial, to listen to the testimony of the common witnesses, and to go into the witness-box and give his expert opinion on the facts proved in evidence? On the one hand it may be said that no one should be compelled against his will to contribute an expression of opinion on the merits of a series of facts in which he has no personal concern; also that the technical equipment of an expert is his own property, and that he need not render this for anyone's benefit except at his own pleasure. On the other hand it may be urged that obedience to a formal legal summons is a necessary duty of citizenship, and that if the law requires for purposes of public justice the opinion of any individual it has a right to demand this. Prominent judges at various times have given conflicting decisions on the point. Hence it is usually considered a safe thing to obey a subpœna even when one's relationship to the cause at issue can only be that of an expert witness. This conclusion will be strengthened by the consequences recently entailed on a professional engineer who declined when duly summoned to attend an inquiry conducted by the Board of Trade into the loss of a trawler. As a result the engineer has been presented with that painful dilemma—a penalty of £5 or a month's imprisonment.

### The Fear of Death.

An Englishman of letters, whose genius and achievements have won for him universal regard, has lately informed his countrymen that "doctors and parsons are doing a lot of harm by increasing the fear of death and making the English less manly." We must leave the "parsons" to stir for themselves, but it may be well to inquire how far the indictment applies to the medical profession. In the first place we venture to doubt the accuracy of the conclusion. That as civilisation has advanced Englishmen, in common with other nations, have become less rough and brutal in their pleasures and their quarrels, and more sensitive to the thought of pain and suffering, need not be denied. It may even be allowed that there is a lessened willingness to bear pain, due partly, we believe, to the greater opportunities for escape and partly to the increased sensitiveness of the nerve centres. But this is a very different conclusion from that phrased by Mr. Meredith. Surely it is not unmanly to avoid pain which is unnecessary either for the welfare of ourself or others. Again it may be admitted that men, perhaps in an increasing degree, shrink from the thought of prolonged illness and incapacity, but this, we take it, is not the fear of death. Indeed we doubt very much whether it is, so far as personal considerations are concerned, very much more than the dread of the loss of the ability to play a manly part in the world. That Englishmen are ready to risk and sacrifice their lives for a great cause recent history vividly shows, and every day records instances of heroic self-sacrifice, unattended by the stimulus of the fighting line. Possibly there is a greater disposition to inquire whether the cause be a good one or a bad one, and this exercise of intelligence seems to us a movement towards true manliness. The wise man, it has been said, fears nothing less than death, and the doctor, with his gospel of courage and his recognition of the inevitable, is an influence in the direction, not of a flabby sentimentality, but towards a lofty and intelligent fortitude.

### Adulteration Made Easy.

An inspector under the Foods and Drugs Act has recently expressed the opinion that the Act is of little use in preventing the cheating of people in the purchase of food. To some extent this may be the purchaser's own fault, for very few of us are absolutely accurate in defining the nature and quality of the commodity we ask for. We name the price we are prepared to pay, and assume that we get good value for the money. No doubt many people think that the Adulteration Act protects them against fraud. To what extent it does so may be estimated from the statement of Dr. Collingridge, the medical officer of health for the City, that there are three districts where the authorities decline to regard as an offence the addition of 7 per cent. of water to the milk sold. But what would the purchasers say if they knew? The fraud is doubtless well concealed, and the price of pure milk is paid for the compound. Coffee, too, is a very convenient article for the fraudulent dealer. Many people have no objection to a little chicory in it; the grocer declares on his bag that what he sells is a mixture of chicory and coffee, and it occurs to no one that the proportion of the former may, as in one case quoted, amount to 80 per cent. of the whole. In any case the tradesman has, by the declaration, saved himself from prosecution. Even though no such printed assurance is given, the dealer can always save himself by the remark that he does not guarantee the article. Regular customers, perhaps, get no such warning, imperfect as it is, if their easygoing content has been tested and proved satisfactory, but a stranger who enters the shop is likely to receive it, especially a stranger who may be suspected of being an inspector. But, indeed, so little in the way of precaution is required to exonerate a tradesman in the eyes of the law that it is surprising that any are ever successfully prosecuted. And until the retailer is compelled to define more clearly what he pretends to sell, and what he actually does sell under the same name, it is to be feared that even if prosecutions are many, convictions will be few.

